# Novel role of miR-29a in pancreatic cancer autophagy and its therapeutic potential

**DOI:** 10.18632/oncotarget.11928

**Published:** 2016-09-10

**Authors:** Jason J. Kwon, Jeffrey A. Willy, Kayla A. Quirin, Ronald C. Wek, Murray Korc, Xiao-Ming Yin, Janaiah Kota

**Affiliations:** ^1^ Department of Medical and Molecular Genetics, Indiana University School of Medicine (IUSM), Indianapolis, IN, USA; ^2^ Department of Biochemistry and Molecular Biology, Indiana University School of Medicine (IUSM), Indianapolis, IN, USA; ^3^ Department of Medicine, Indiana University School of Medicine (IUSM), Indianapolis, IN, USA; ^4^ The Melvin and Bren Simon Cancer Center, Indiana University School of Medicine (IUSM), Indianapolis, IN, USA; ^5^ Center for Pancreatic Cancer Research, Indiana University and Purdue University-Indianapolis (IUPUI), Indianapolis, IN, USA; ^6^ Department of Pathology and Laboratory Medicine, Indiana University School of Medicine (IUSM), Indianapolis, IN, USA

**Keywords:** pancreatic cancer, autophagy, gemcitabine, metastasis, miR-29

## Abstract

Pancreatic Ductal Adenocarcinoma (PDAC) is a highly lethal malignancy that responds poorly to current therapeutic modalities. In an effort to develop novel therapeutic strategies, we found downregulation of miR-29 in pancreatic cancer cells, and overexpression of miR-29a sensitized chemotherapeutic resistant pancreatic cancer cells to gemcitabine, reduced cancer cell viability, and increased cytotoxicity. Furthermore, miR-29a blocked autophagy flux, as evidenced by an accumulation of autophagosomes and autophagy markers, LC3B and p62, and a decrease in autophagosome-lysosome fusion. In addition, miR-29a decreased the expression of autophagy proteins, TFEB and ATG9A, which are critical for lysosomal function and autophagosome trafficking respectively. Knockdown of TFEB or ATG9A inhibited autophagy similar to miR-29a overexpression. Finally, miR-29a reduced cancer cell migration, invasion, and anchorage independent growth. Collectively, our findings indicate that miR-29a functions as a potent autophagy inhibitor, sensitizes cancer cells to gemcitabine, and decreases their invasive potential. Our data provides evidence for the use of miR-29a as a novel therapeutic agent to target PDAC.

## INTRODUCTION

Pancreatic Ductal Adenocarcinoma (PDAC) is one of the most lethal forms of human malignancies worldwide with poor prognoses [1]. In the United States, PDAC is the fourth leading cause of cancer related deaths [2] and is projected to become the second leading cause of cancer deaths by 2030 [3]. PDAC is often undiagnosed until it has metastasized and these advanced tumors display resistance to existing therapeutic modalities. Although there have been recent improvements in combination chemotherapies such as Nab-Paclitaxel/Gemcitabine and FOLFIRINOX [4, 5], the overall 5-year survival rate has not exceeded 8% [2]. Furthermore, PDAC has a well-characterized mutational profile that plays a key role in disease onset and progression (>90% cases with KRAS mutations and >50% with inactivating mutations in p53, CDKN2A, or SMAD4) [6], but the knowledge of these genetic perturbations has yet to yield targeted therapies. The lack of effective treatments and early detection necessitates the critical need to further dissect molecular mechanisms associated with PDAC progression to develop novel and effective therapeutic strategies for improving patient survival.

Macroautophagy, herein referred to as autophagy, is the process in which cells degrade internal constituents for the maintenance of cellular homeostasis and survival under stress conditions [7]. When autophagy is induced, cytoplasmic components are sequestered into double-membrane vesicles called autophagosomes, which then fuse with lysosomes. Subsequently, the hydrolases of the lysosomal compartments degrade cytoplasmic cargo and release the basic cellular building blocks into the cytosol for recycling [7]. Recent studies document that the upregulation of autophagy can serve as a survival mechanism in various malignancies [8–19], including PDAC tumor growth and progression [15–17]. These reports have paved the way for clinical trials utilizing hydroxychloroquine (HCQ), a lysosomotropic agent, in PDAC patients to inhibit autophagy as a means of therapy (clinicaltrials.gov NCT01273805). However, HCQ is associated with toxicity and off-target effects such as neuromyotoxicity [20], retinopathy [21, 22], and cardiomyopathy [23, 24].

Increasing evidence suggests that microRNA (miRNA)-based therapeutics have limited off-target effects and could emerge as novel therapeutic agents for various human diseases including cancer [25–31]. miRNAs are conserved small non-coding RNAs that regulate post-transcriptional gene expression [32, 33]. These small molecules are abundantly expressed in normal tissue, and are often misregulated in disease states. Restored expression of downregulated miRNAs has been suggested to be beneficial in therapeutically targeting cancer [34–36]. We and others have recently found miR-29 to be downregulated in PDAC [37–39]. Of importance, overexpression of miR-29 in stromal cells reduced the accumulation of stromal proteins and cancer colony formation in direct co-cultures [39].

In this study, we address the role of miR-29 in pancreatic cancer cells. We found downregulation of miR-29 in a range of pancreatic cancer cell lines, and restored expression of miR-29a blocked autophagy flux by inhibiting expression of key autophagy proteins, TFEB and ATG9A. Furthermore, miR-29a overexpression sensitized chemoresistant pancreatic cancer cells to gemcitabine and reduced their invasive potential. Our findings provide evidence for the use of miR-29a as a novel therapeutic agent to target PDAC.

## RESULTS

### miR-29a sensitizes chemotherapeutic resistant pancreatic cancer cell lines to gemcitabine treatment

**Figure 1:**
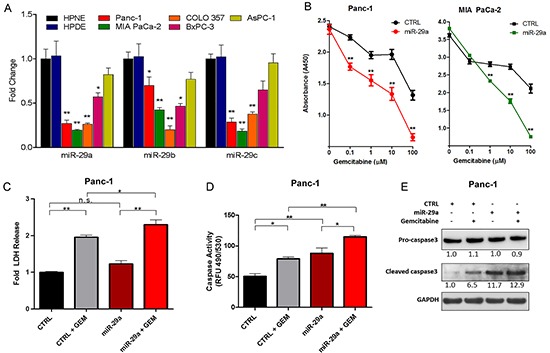
miR-29a increased sensitivity of chemoresistant PDAC cells to gemcitabine treatment **A.** qPCR analysis of miR-29 family members in normal human ductal epithelial cell lines (HPNE and HPDE) and pancreatic cancer cell lines (Panc-1, MIA PaCa-2, COLO 357, BxPC-3, AsPC-1) (n=4). Data represented as average fold change (^ΔΔ^CT) ± standard error of the mean (S.E.M.) **B.** Pancreatic cancer cell lines (Panc-1 and MIA PaCa-2) were seeded into 96-well plates, transfected with control (CTRL) or miR-29a mimics, treated with indicated concentrations of gemcitabine (GEM) for 24 hours post-transfection, and viability was measured at 72 hours post-treatment using the Cell Counting Kit-8 (CCK-8). Average absorbance (A450) is represented (n=8) ± S.E.M. **C.** Panc-1 cells were transfected with CTRL or miR-29a mimics, treated with 10μM GEM for 48 hours and lactate dehydrogenase (LDH) release was determined by substrate based activity assay (fluorescence 560/590nm). Average relative percent cytotoxicity are represented (n=3-4) ± S.E.M. **D.** Panc-1 cells were transfected with CTRL or miR-29a mimics. 24 hours post-transfection cells were treated with 10μM GEM for 24 hours, lysed, and caspase activity was determined by absorbance using Apo-ONE Homogeneous Caspase-3/7 Assay according to manufacturer's protocol. Average relative fluorescence (RFU, 490/530nm) are represented (n=4) ± S.E.M. **E.** Panc-1 transfected with CTRL or miR-29a mimics, treated with 10μM GEM for 12 hours and 15ug of total cell protein lysate was subjected to western blot analysis for procaspase-3, cleaved caspase-3, and GAPDH was used as loading control. Relative quantification of band intensities normalized to GAPDH are shown below respective blots. All experiments were repeated 3-4 times and representative data is presented. *p<0.05, **p<0.01, non-significant (n.s.).

Previously, we observed a global and epithelial-specific decrease in miR-29 expression in the pancreata of a well-characterized pancreatic cancer mouse model, LSL-KRas^G12D^; Pdx1Cre, and human PDAC patients [39]. To understand the function of miR-29 in pancreatic cancer cells, we initially measured the expression levels of miR-29 in five pancreatic cancer cell lines (Panc-1, MIA PaCa-2, COLO 357, BxPC-3, and AsPC-1) compared to two normal human pancreatic ductal epithelial cell lines (HPNE and HPDE). There was a significant decrease in miR-29a and miR-29b expression in four out of five pancreatic cancer cell lines compared to normal human pancreatic ductal epithelial cells and levels of miR-29c were lower in three out of five pancreatic cancer cell lines (Figure 1A). We have previously reported that miR-29a is the most abundantly expressed miR-29 family member in the human pancreas and in pancreatic stellate cells [39] and also found that miR-29a is the most highly expressed miR-29 family member in the normal human pancreatic epithelial cell line, HPNE (Supplementary Figure S1). Therefore, we focused on miR-29a for functional studies.

One of the major limitations in treating PDAC is that cancer cells acquire resistance to existing chemo and radiation therapeutic modalities, including gemcitabine [40]. Furthermore, pancreatic cancer cells are surrounded by dense fibrotic stroma, which is known to impair drug delivery to the tumor core [41]. Previously, we showed that overexpression of miR-29a in pancreatic stellate cells, the major stromal cells responsible for fibrotic stroma, reduced extracellular matrix protein accumulation and cancer growth in co-cultures, suggesting its potential use as a novel therapeutic agent in normalizing stromal abundance to target PDAC [39]. To test the feasibility of combining miR-29a with gemcitabine, a standard therapy, we investigated the effect of miR-29a overexpression on viability of known gemcitabine resistant pancreatic cancer cell lines, Panc-1 and MIA PaCa-2 [42]. Overexpression of miR-29a alone did not significantly reduce the viability of cancer cells, but the addition of gemcitabine resulted in a significant decrease at various concentrations starting at 0.1μM (Figure 1B). Furthermore, miR-29a did not have any additive effect on cell viability in gemcitabine sensitive PDAC cell line, COLO 357 [43], to drug treatment (Supplementary Figure S2).

Evasion of cell death is a crucial event during malignant transformation of pancreatic cancer cells. Initially, we determined the cytotoxic effects of miR-29a in combination with gemcitabine on PDAC cell lines resistant to gemcitabine (Panc-1 and MIA PaCa-2) by measuring the release of lactate dehydrogenase (LDH) [44, 45]. There was a significant increase in LDH from the Panc-1 and MIA PaCa-2 cells overexpressing miR-29a upon gemcitabine treatment, indicating that miR-29a increases cytotoxicity in combination with gemcitabine (Figure 1C and Supplementary Figure S3). We also observed increased caspase-3/7 activity (Figure 1D and Supplementary Figure S4A) and cleaved caspase-3 levels (Figure 1E and Supplementary Figure S4B) in miR-29a overexpressing cancer cells upon gemcitabine treatment compared to cancer cells treated with gemcitabine alone. Taken together, these findings indicate that miR-29a sensitizes pancreatic cancer cells to gemcitabine treatment and provides compelling evidence for its use in combination with gemcitabine as a novel therapeutic strategy to target PDAC.

### miR-29a inhibits autophagy flux in pancreatic cancer cells

Pancreatic cancer cells induce autophagy as a survival mechanism to escape gemcitabine induced cell death [17–19]. Therefore, we sought to determine the effect of miR-29 on autophagy, to determine whether the increased sensitivity and cytotoxic effects of gemcitabine in chemotherapeutic resistant pancreatic cancer cells is due to alterations in autophagy. LC3B is a widely used marker to monitor autophagy levels [46]. Normally, LC3B resides in the cytoplasm (LC3BI), and upon initiation of autophagy, it is conjugated with phosphatidyl-ethanolamine (LC3BII) to facilitate formation and expansion of the autophagosome membrane [47–51].

**Figure 2:**
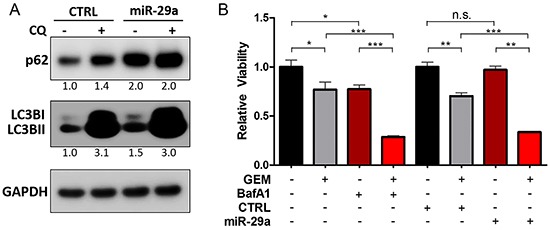
miR-29a overexpression causes blockage in autophagy flux **A.** Panc-1 cells were transfected with CTRL or miR-29a mimics. Following transfection, cells were treated with 25μM Chloroquine (CQ) and 5ug of total cell lysates were subjected to western blot analysis for p62 and LC3B, and GAPDH was used as loading control. Relative quantification of band intensities normalized to GAPDH are shown below respective blots. **B.** Panc-1 cells were transfected with CTRL or miR-29a mimics and treated with and without 10μM GEM. In parallel, Panc-1 cells were treated with 10μM GEM alone or in combination with 10μM BafA1. 48 hours post GEM treatment, viability was determined using CCK-8 assay kit. Average relative absorbance (A450) normalized to respective controls is presented (n=6) ± S.E.M. All experiments were repeated 3-4 times and representative data is presented. *p<0.05, **p<0.01, and ***p<0.001.

To elucidate the effects of miR-29a on PDAC autophagy, Panc-1 cells, which have high basal levels of autophagy [17], were transfected with miR-29a or mimic control and LC3B levels were assessed by western blot analysis. There was a marked increase in LC3B upon miR-29a overexpression in Panc-1 cells (Figure 2A). Similar observations were found in MIA PaCa-2 and COLO 357 cells (Supplementary Figure S5A and S5B). An increase in LC3B levels can indicate an upregulation of autophagy or a blockage of autophagy flux [52]. We therefore examined the expression of an autophagic substrate, p62, in conjunction with LC3B. p62/SQSTM1 is efficiently degraded upon autophagy induction and serves as an indicator of autophagic turnover [52]. An increase in p62 levels correlates with an inhibition in autophagy, whereas a decrease indicates induction of autophagy [53]. There was a robust accumulation of p62 in miR-29a overexpressing cancer cells (Figure 2A, Supplementary Figure S5A, and S5B), suggesting that miR-29a causes a late stage blockage in autophagy flux. It is possible that the increase in p62 could be due to an indirect transcriptional upregulation rather than inhibition of autophagy. Therefore, we measured p62 transcript levels and found no significant change (Supplementary Figure S6), indicating that the p62 accumulation is due to a perturbation in autophagy.

Chloroquine (CQ) is a lysosomotropic agent that inhibits autophagy by raising lysosomal pH [54]. The increase in lysosomal pH permits autophagosome-lysosome fusion but prevents degradation and autolysosomal turnover [55]. To further assess the effect of miR-29a on autophagy flux, miR-29a or mimic control was transiently expressed in Panc-1 cells and then treated with CQ. Our results showed a net increase in LC3BI and II and p62 accumulation in control cells upon CQ addition. However, when miR-29a is overexpressed, prior to CQ treatment, the net difference of LC3B and p62 between miR-29a alone compared to miR-29a and CQ combination treatment was low, as miR-29a had already blocked autophagy (Figure 2A). These results were further recapitulated in MIA PaCa-2 and COLO 357 cells (Supplementary Figure S5A and S5B). Similar to CQ, BafilomycinA1 (BafA1) functions as a late stage inhibitor of autophagy by raising lysosomal pH [56] and also blocks autophagosome-lysosome fusion [57]. Treatment of Panc-1 and MIA PaCa-2 with other late stage autophagy inhibitors, CQ and BafA1, resulted in p62 and LC3B accumulation similar to miR-29a overexpression (Supplementary Figure S7).

Pancreatic cancer cells have been previously shown to induce autophagy and acquire resistance to chemotherapy [16, 58, 59]. To verify the functional effect of miR-29a mediated blockage of autophagy flux on gemcitabine sensitization, we evaluated the effects of miR-29a on cancer cell viability in comparison with BafA1. Similar to miR-29, treatment of cancer cells with gemcitabine in combination with BafA1 decreased cancer cell viability (Panc-1 and MIA PaCa-2) compared to gemcitabine alone (Figure 2B and Supplementary Figure S8). To further elucidate the effect of miR-29a and gemcitabine combination on pancreatic cancer cell autophagy, miR-29a expressing Panc-1 and MIA PaCa-2 cells were treated with gemcitabine and subjected to LC3B and p62 western blot analysis (Supplementary Figure S9). Similar to previous reports, Panc-1 and MIA PaCa-2 cells treated with gemcitabine alone exhibited a marked increase in LC3BII and decrease in p62, indicating autophagy induction. However, miR-29a overexpressing cancer cells did not exhibit any net decrease in p62 with an increase in LC3B levels upon gemcitabine treatment due to a miR-29a mediated inhibition of autophagy (Supplementary Figure S9). Taken together, these findings suggest that miR-29a functions as a late stage autophagy inhibitor and sensitizes chemoresistant pancreatic cancer cell lines (Panc-1 and MIA PaCa-2) to gemcitabine treatment.

### miR-29a inhibits autophagosome-lysosome fusion

**Figure 3:**
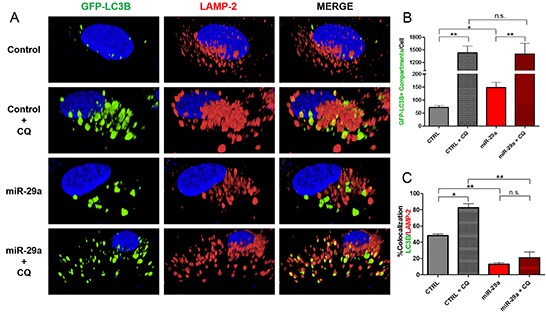
miR-29a inhibits autophagosome-lysosome fusion **A.** Panc-1 stably expressing GFP-LC3B were transfected with CTRL or miR-29a mimics. Following transfection, cells were treated with 25μM CQ. Cells were fixed and stained for lysosomal-associated membrane protein 2 (LAMP-2). **B.** Image analysis was conducted to quantify number of GFP-LC3B positive compartments per cell, and averages are presented ± S.E.M. **C.** Colocalization was calculated based on GFP-LC3B and LAMP-2 staining, and average percentage of colocalization is presented ± S.E.M. Experiment was repeated 3 times and representative data is presented. *p<0.05, **p<0.01, non-significant (n.s.).

During autophagy, autophagosomes fuse with lysosomes where lysosomal hydrolases degrade autophagosomal contents to be recycled back into the cytoplasm [7]. To understand the mechanisms by which miR-29a mediates blockage of autophagy flux, we evaluated its impact on autophagosomes and their interactions with lysosomes. Panc-1 cells stably expressing GFP-LC3B were transfected with miR-29a or control mimics, treated with 25μM CQ, and stained with lysosomal-associated membrane protein 2 (LAMP-2), a lysosomal marker. In subsequent image analysis, we observed a two-fold increase in accumulation of autophagosomes/autophagolysosomes in miR-29a overexpressing cells (Figure 3A, 3B, and Supplementary Figure S10). Furthermore, overexpression of miR-29a resulted in a >35% decrease in LC3B/LAMP-2 colocalization at basal levels and >60% decrease in miR-29a overexpressing cells treated with CQ, compared to CQ alone (Figure 3A, 3C, and Supplementary Figure S11), indicating miR-29a mediated blockage of autophagosome-lysosome fusion.

### miR-29a downregulates critical autophagy proteins TFEB and ATG9A

**Figure 4:**
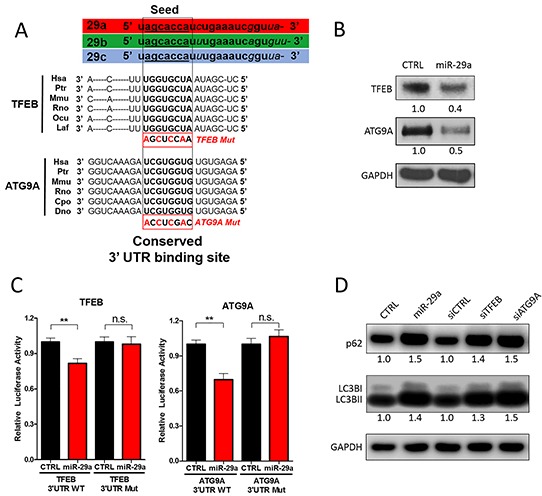
miR-29a downregulates TFEB and ATG9A to inhibit autophagy **A.** Schematic representation of the miR-29 family members and 3′-UTR binding sites of miR-29 targets as well as mutated binding sites used in Luciferase Assays: Transcription Factor EB (TFEB) and Autophagy-related protein 9A (ATG9A). All three miR-29 family members (miR-29a, miR-29b, and miR-29c) have identical seed sequences. Conserved miR-29 binding sites in the 3′-UTR of mRNA transcripts encoding ATG9A and TFEB are depicted in bold. **B.** 10ug of total protein cell lysates from Panc-1 transfected with CTRL or miR-29a mimics were subjected to western blot analysis for TFEB, ATG9A, and GAPDH. Relative quantification of band intensities normalized to GAPDH are shown below respective blots. **C.** Relative firefly luciferase activity from TFEB and ATG9A 3′ UTR wild type (WT) and mutant (mut) reporter constructs following co-transfection into Panc-1 cells with control or miR-29a mimics. All readouts were normalized to renilla luciferase activity for each well. Average relative luminesce normalized to respective controls is presented (n=6) ± S.E.M. **D.** 5ug of total protein cell lysates from Panc-1 cells were transfected with CTRL, miR-29a mimics, siCTRL, siTFEB, or siATG9A. 24 hours post-transfection, total protein was harvested and subjected to western blot analysis for p62 and LC3B, and GAPDH was used as loading control. Quantification of band intensities normalized to GAPDH and relative to control are shown below respective blots. All experiments were repeated 3 times and representative data is presented.

To address the mechanisms by which miR-29a mediates blockage of autophagy at late stages, we searched for potential autophagy related genes that are targeted by miR-29a using four prediction algorithms (TargetScan, PicTar, PITA, and miRanda). We found that both transcription factor EB (*TFEB*) and autophagy-related protein 9A (*ATG9A*) contain phylogenetically conserved miR-29 binding sites in their 3′-UTRs (Figure 4A). TFEB is a transcription factor and member of the MiT/TFE family, which has been shown to be an integral part of the lysosome and autophagy machinery [60]. Upregulation of MiT/TFE factors has been implicated in various cancers [61–63], and a recent study documented that upregulation of TFEB contributes to increased autophagy in PDAC [63]. Furthermore, knockdown of TFEB impaired growth and metabolism of PDAC cells by disrupting lysosomal catabolism in autophagy [63]. Among >30 essential autophagy-related (ATG) genes [64], ATG9A is the only transmembrane protein [65] and has been shown to facilitate trafficking lipid membrane from the Golgi network and endosomes to the formation of autophagosomes [66, 67]. ATG9A has been shown to be upregulated in some carcinomas [68, 69] but has yet to be studied in PDAC. In our western blot analysis, overexpression of miR-29a in pancreatic cancer cells resulted in a marked downregulation of both TFEB and ATG9A expression (Figure 4B and Supplementary Figure S12). As miRNAs regulate the expression of multiple target mRNAs, to test whether miR-29a regulates expression of GAPDH, an endogenous control, we conducted target prediction analysis (Targetscan) and confirmed that GAPDH is not a target of miR-29a (data not shown), ensuring its use as a proper loading control in our western blot analysis.

As we observed downregulation of TFEB and ATG9A with miR-29a overexpression, to verify whether miR-29a directly regulates their expression, TFEB and ATG9A 3′UTR luciferase reporter plasmids were developed for regions containing miR-29 predicted binding sites. Both wild type and mutated binding sites (Figure 4A) were cloned into the 3′UTR downstream of the luciferase open reading frame. When reporter plasmids with wild type miR-29a binding sites were co-transfected with miR-29a mimics into cancer cells, we found a significant repression of luciferase activity. However, when the 3′UTRs were mutated, miR-29a no longer has the ability to repress luciferase activity of both TFEB and ATG9A, demonstrating that miR-29a represses TFEB and ATG9A expression by directly interacting at the predicted sites. Consistently, we observed higher ATG9A and TFEB expression in pancreatic cancer cells that have low miR-29a expression (Panc-1 and MIA PaCa-2), compared to normal pancreatic epithelial cell line (HPNE) and cancer cell line with high miR-29 expression (AsPC-1) (Supplementary Figure S13). Taken together, our data implicates that miR-29a downregulates TFEB and ATG9A through direct interactions with the 3′UTR binding sites.

**Figure 5:**
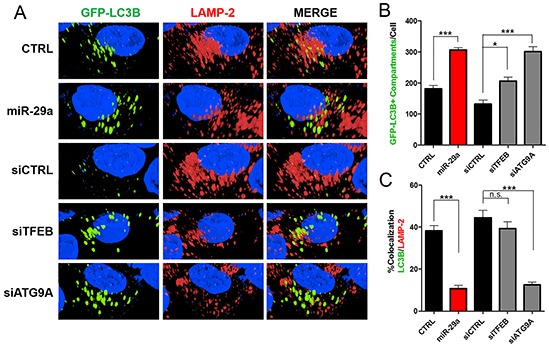
Knockdown of TFEB and ATG9A results in decreased autophagosomal-lysosomal fusion **A.** Panc-1 cells stably expressing GFP-LC3B were transfected with either CTRL or miR-29a mimics in parallel to cells transfected with siCTRL, siTFEB, or siATG9A. Following transfection, cells were fixed and stained for lysosomal-associated membrane protein 2 (LAMP-2). **B.** Image analysis was conducted to quantify number of GFP-LC3B positive compartments per cell, and averages are presented ± S.E.M. **C.** Colocalization was calculated based on GFP-LC3B and LAMP-2 staining, and average percentage of colocalization is presented ± S.E.M. All experiments were repeated 3 times and representative data is presented. **p<0.05, **p<0.01, ***p<0.001 non-significant (n.s.).

Next, we sought to determine the effects of TFEB and ATG9A depletion on PDAC autophagy using siRNA mediated knockdown of TFEB or ATG9A. Knockdown of these two genes resulted in an accumulation of LC3B and p62 similar to miR-29a overexpression (Figure 4D and Supplementary Figure S14). Furthermore, knockdown of TFEB and ATG9A led to a significant increase in accumulation of GFP-LC3B positive vesicles (Figure 5). TFEB knockdown resulted in a ~50% increase of autophagosome accumulation (Figure 5A and 5B), whereas ATG9A knockdown caused a >100% increase in autophagosome accumulation (Figure 5A and 5B). Although knockdown of TFEB blocked autophagy, as indicated by an increased accumulation in p62 and LC3B (Figure 4C), we did not find a significant difference in GFP-LC3B and LAMP-2 colocalization, suggesting that the increase in GFP-LC3B positive vesicles were mostly due to accumulation of autophagolysosomes (Figure 5A and 5B). Whereas, knockdown of ATG9A resulted in a robust 2-fold decrease in colocalization of LC3B and LAMP-2, demonstrating that miR-29a inhibits autophagosome-lysosome fusion predominately by deregulation of ATG9A. Taken together, our results suggests that miR-29a inhibits autophagy flux through the downregulation of TFEB and ATG9A expression, which are critical for lysosomal function and autophagosome trafficking respectively.

### miR-29a reduces pancreatic cancer cell invasion *in vitro*

**Figure 6:**
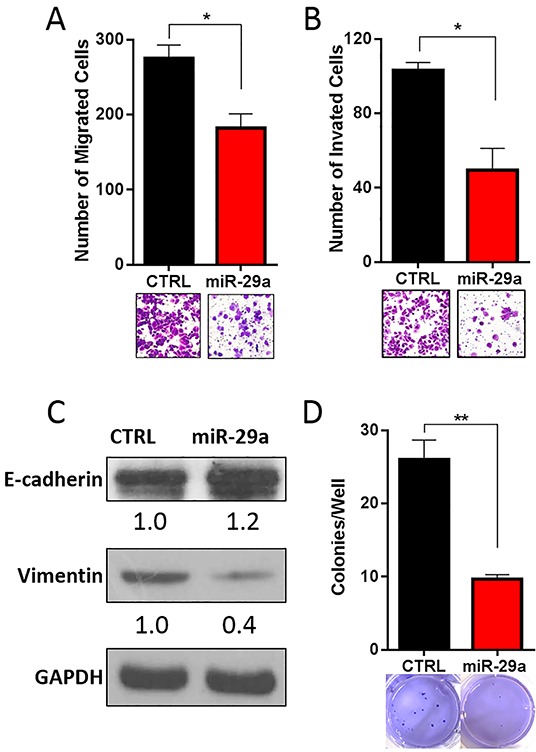
miR-29a inhibits invasive potential of PDAC cells Panc-1 cells were transfected with control (CTRL) or miR-29a and plated into **A.** migration, and **B.** invasion assays. Migration and invasion data presented as average number of cells per 5 fields (n=3) ± S.E.M. and representative images shown below each graph. **C.** 10ug of total cell lysates from Panc-1 transfected with CTRL or miR-29a mimics were subjected to western blot analysis for epithelial marker, E-cadherin, and mesenchymal marker, Vimentin, and GAPDH was used as loading control. Relative quantification of band intensities normalized to GAPDH are shown below respective blots. **D.** Panc-1 cells were transfected with CTRL or miR-29a mimics and plated into soft agar assays. Data presented as average number of colonies per well (n=6) ± S.E.M. and representative images are shown below each graph. All experiments were repeated 3-4 times and representative data is presented. *p<0.05, **p<0.01.

Prior studies have also found that miR-29 is downregulated in a wide variety of carcinomas, including breast, colorectal, and prostate, and its reintroduction had anti-metastatic effects [70–72]. Furthermore, increase in autophagy has been shown to enhance the invasive potential of cancer cells and promote epithelial-mesenchymal transition (EMT) [11, 12]. As we observed downregulation of miR-29 in pancreatic cancer cells and its subsequent overexpression inhibited autophagy, we performed a series of *in vitro* functional studies to evaluate the effect of miR-29a on the invasive potential of pancreatic cancer cells. To determine the effect of miR-29a on cancer cell migration and invasion, Panc-1 and MIA PaCa-2 cells were transfected with control or miR-29a mimics and seeded in transwell assays. Compared to control cells, significantly fewer miR-29a overexpressing cancer cells migrated through transwell membranes and invaded through matrigel-precoated membranes (Figure 6A, 6B, Supplementary Figure S15A, and S15B).

EMT is known to enhance the migration/invasion of pancreatic cancer cells and resistance to apoptosis [73–75]. As miR-29a reduced the migration and invasion potential of pancreatic cancer cells, we sought to determine its effect on EMT. As expected, overexpression of miR-29a in pancreatic cancer cells increased expression of epithelial marker, E-cadherin [74] and decreased mesenchymal marker, Vimentin [74] (Figure 6C).

We next tested the effect of miR-29a overexpression on anchorage independent growth of pancreatic cancer cells using soft agar assays. There was a significant decrease in the number of anchorage independently growing cancer colonies in miR-29a overexpressing PDAC cells compared to cells expressing control mimic (Figure 6D and Supplementary Figure S16). Our data demonstrates the anti-invasive potential of miR-29a evidenced by a reduction in migration, invasion, and anchorage independent growth of pancreatic cancer cells.

## DISCUSSION

**Figure 7:**
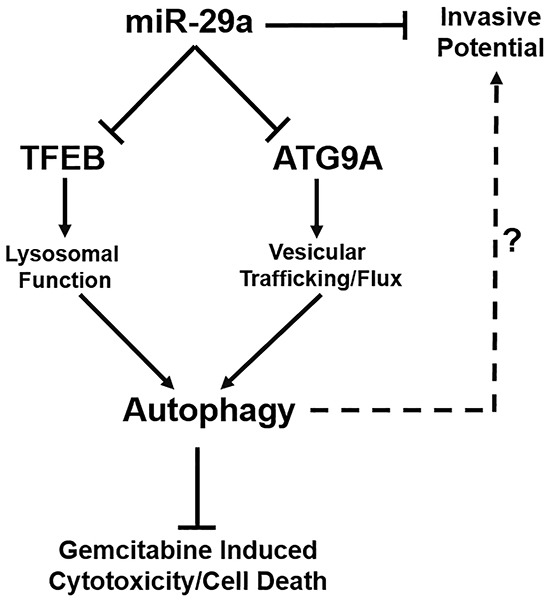
Schematic diagram representing the role of miR-29a in PDAC autophagy and metastasis miR-29a overexpression in pancreatic cancer cells decreases invasive potential and inhibits autophagy flux through downregulation of TFEB and ATG9A, resulting in increased sensitivity to GEM treatment. miR-29a may serve as a potential novel anti-autophagic/invasive agent to target PDAC.

For the first time, we demonstrate that miR-29a functions as a potent autophagy inhibitor, sensitizes chemoresistant cancer cells to gemcitabine, and reduces the invasive potential of pancreatic cancer cells (Figure 7). Increasing evidence documents the role of autophagy in cancer pathogenesis, and has proven to be a novel therapeutic target [8–17]. We found consistent downregulation of miR-29 in pancreatic cancer cells, and its overexpression inhibited autophagy, evidenced by increased accumulation of autophagosomes/autophagolysosomes and autophagy markers, LC3B and p62. Furthermore, miR-29a decreased autophagosome-lysosome fusion, as evidenced by a significant decrease in colocalization of LC3B and LAMP-2, autophagosomal and lysosomal markers respectively. Taken together, our results suggest that miR-29a functions as a late stage autophagy inhibitor and blocks autophagosome-lysosome fusion.

To determine the mechanisms of miR-29a mediated inhibition of autophagy, we identified two critical autophagy proteins, TFEB and ATG9A, which have phylogenetically conserved miR-29 binding sites in their 3′-UTRs. As expected, overexpression of miR-29a caused a marked reduction of TFEB and ATG9A expression. TFEB, a transcription factor essential for lysosomal function, is highly activated in PDAC [60] and its knockdown reduces tumor progression and impairs autophagy due to lysosomal dysfunction [63]. ATG9A is the only transmembrane ATG protein, and facilitates membrane trafficking of autophagosomes [76, 77]. Upregulation of ATG9A has been well documented in other carcinomas [68, 69]. Knockdown of TFEB or ATG9A caused a late stage blockage in autophagy similar to miR-29a overexpression. Interestingly, knockdown of ATG9A or TFEB increased the accumulation of LC3B positive compartments, but only the knockdown of ATG9A blocked autophagosome-lysosome fusion. As ATG9A functions in vesicular trafficking, reduced expression of ATG9A is likely to cause perturbations in autophagosome trafficking and prevent them from fusing with lysosomes. Whereas, TFEB knockdown mediated accumulation of autophagolysosomes is likely due to defective lysosomal degradation capacity rather than a blockage of autophagosome-lysosome fusion. Collectively, our data indicates that miR-29a inhibits PDAC autophagy by downregulation of TFEB and ATG9A.

Pancreatic cancer acquires chemo-resistance by inducing autophagy [15–17]. We found that miR-29a sensitized chemoresistant pancreatic cancer cells to gemcitabine treatment, decreased cancer cell viability, and enhanced gemcitabine-mediated cytotoxicity. Accordingly, upon gemcitabine treatment, miR-29a overexpression resulted in an increased LDH release [44, 45], caspase 3/7 activity, and cleaved caspase 3. CQ and BafA1 are known late stage autophagy inhibitors [54, 57]. The effect of miR-29a on LC3B and p62 accumulation is similar to CQ and BafA1, suggesting that miR-29a serves as an effective late stage inhibitor of autophagy. miR-29 has been previously reported to induce cellular cytotoxicity/apoptosis by targeting anti-apoptotic protein Mcl-1 in cholangiocarcinoma [78]. However, the effect of miR-29 on Mcl-1 and gemcitabine-mediated cytotoxicity is not known in pancreatic cancer cells. Further studies are required to dissect the role of Mcl-1 in this mechanistic axis.

Our functional studies demonstrate that overexpression of miR-29a inhibits pancreatic cancer cell migration and invasion through reversion of cancer cell EMT. Prior to metastasis, tumor cells undergo EMT, a process in which cells lose epithelial characteristics and gain a mesenchymal phenotype. Recent reports have implicated EMT is a characteristic feature of malignant transformation that contributes to the invasive/metastatic properties of a wide variety of cancers [79]. Overexpression of miR-29a resulted in an increase in epithelial marker, E-cadherin, and reduction in mesenchymal marker, Vimentin, indicating that reintroduction of miR-29a induces mesenchymal–epithelial transition (MET) in pancreatic cancer cells. However, Vimentin is not a predicted miR-29 target (Targetscan), suggesting that miR-29a may induce MET transition through indirect effects rather than direct regulation of Vimentin. Of interest, autophagy has been shown to be concomitant with increased metastasis and EMT [11, 12]. Our findings demonstrate similar effects in which miR-29a mediated an inhibition of autophagy in tandem with MET transition and reduction of cancer cell invasive potential. It is possible that miR-29a reduces EMT in pancreatic cancer cells by inhibition of autophagy. However, further studies are warranted to dissect the precise underlying mechanisms.

To further study the function of miR-29a, we investigated the effect of miR-29a on anchorage independent growth of pancreatic cancer cells. Epithelial cells require attachment to a substrate in order to maintain structural polarity, function, and survival [80]. When this adhesion is lost, cells undergo anoikis, a form of programmed cell death induced by detachment [80, 81]. Various cancers have been shown to avoid anoikis, leading to properties associated with metastasis and tumorigenesis [82]. We found that overexpression of miR-29a significantly reduces the ability of pancreatic cancer cells to grow in an anchorage independent manner.

With the exception of AsPC-1, we observed consistent downregulation of all three miR-29 family members in various pancreatic cancer cell lines. AsPC-1 contains homologous deletions in SMAD4, whereas the other cancer cell lines contain wildtype SMAD4 [83]. SMAD4 is a major downstream effector molecule of TGF-β1 signaling, forms a heterogeneous complex with pSMAD2/3, and regulates the target gene expression [84]. We and others have previously demonstrated that TGF-β1 downregulates miR-29 expression in other cell types through SMAD2/3/4 complex [39, 85]. Perhaps homologous deletion of SMAD4 in AsPC-1 derepresses miR-29 expression. However, this mechanism needs to be validated in PDAC. Furthermore, the regulatory mechanism associated with downregulation of miR-29 has not been fully elucidated. The three miR-29 family members (miR-29a, -29b, -29c) are encoded from two co-transcribed miRNA clusters, miR-29a/b1 and 29c/b2 located on Chromosome 7 and 1 respectively. Both miR-29 loci have promoter regions containing c-Myc [86] and SMAD3/4 [85] binding sites. It would be of interest to determine if c-Myc or SMAD4 regulates miR-29 expression in pancreatic cancer cells, as both of them have been indicated to play a critical role in PDAC [87, 88].

The downregulation and anti-tumorigenic effects of miR-29 have been reported in a wide variety of cancers [70–72]. Furthermore, autophagy has been implicated in a number of carcinomas to promote metastasis [9] and induce chemoresistance [89–91]. It would be of immense interest to evaluate the role of miR-29 in autophagy of other cancers, as this would shed light on whether the mechanism of autophagy inhibition we have found is ubiquitous in cancer pathogenesis or idiosyncratic to pancreatic cancer. Furthermore, our findings need to be validated *in vivo* to translate our findings into the clinic. Nevertheless, our studies reveal a novel role of miR-29a in PDAC autophagy and provide evidence for its use as an autophagy inhibitor and novel therapeutic agent in combination with gemcitabine to target PDAC.

## MATERIALS AND METHODS

### Cell lines

Normal human pancreatic epithelial cell lines HPNE (ATCC, CRL-4023) and HPDE (AddexBio, T0018001) were grown in Dulbecco's Modified Eagle Medium (DMEM) (Life Technologies, 11965-092) supplemented with 10% fetal bovine serum (FBS). Panc-1 (ATCC, CRL-1469) and MIA PaCa-2 (ATCC, CRL-1420) were grown in DMEM supplemented with 10% FBS, 100units ml^−1^ penicillin, and 100mg ml^−1^ streptomycin. COLO 357 [92], AsPC-1 (ATCC, CRL-1682), and BxPC-3 (ATCC, CRL-1687) were grown in Roswell Park Memorial Institute (RPMI) 6140 (Life Technologies, 11875-093) supplemented with 10% FBS, 100units ml^−1^ penicillin, and 100mg ml^−1^ streptomycin.

### RNA purification

Total RNA was extracted from cells using Trizol extraction kit (Life Technologies, 15596018) according to the manufacturer's protocol. The quantity and purity of RNA was determined by OD260/280 reading using a Nanodrop spectrophotometer.

### Measurements of RNA by qPCR

Mature miR-29 family member expression and p62 mRNA expression levels were measured by TaqMan Assays (Applied Biosystems): miR-29a (ID:002112); miR-29b (ID:000413); and miR-29c (ID:000587) ; and SQSTM1/p62 (ID: 4331182). U6 snRNA (ID:001973) or ACTB (ID: 4331182) were used as a endogenous controls to normalize miR-29 expression and p62 expression respectively. Samples were analyzed using ABI 7500 Real-Time PCR machine. Samples were run in triplicates with 0.2 thresholds, and the ^ΔΔ^CT method was used for relative miR-29 expression analysis.

### Western blot analyses of proteins

Total cell protein was isolated using RIPA buffer (Thermo Scientific, PI-89900) and quantified using BCA Protein Assay Kit (Pierce Biotechnology, 23225). Protein samples were run through SDS-PAGE and were transferred to polyvinylidene fluoride membrane, followed by a block in 10% dried non-fat milk, and then probed with primary antibodies against Caspase-3 (Novus Biological, 9662S), Procaspase-3 (Cell Signaling, 9662S), LC3B (Novus Biological, NB100-2220), SQSTM1/p62 (Thermo Scientific, H00008878-M01), LAMP-2 (Santa Cruz, sc-18822), ATG9A (ab108338), TFEB (Cell Signaling, 4240), GAPDH (Millipore, MAB374) and corresponding HRP conjugated goat anti-rabbit (Santa Cruz, sc-2004), goat anti-mouse (Bio-Rad, 172-1011), or donkey anti-goat (Santa Cruz, sc-2020) secondary antibodies. Proteins were visualized and quantified using chemiluminescent detection kit (GE Healthcare, Amersham ECL) and exposed to X-ray film (Thermo Scientific, CL-X Posure Film) or captured on an Amersham Imager 600 (GE Healthcare, CCD Model). The intensity for each band was densitometrically quantified and normalized against loading control using ImageJ software.

### Transfection of cultured cells

Exponentially growing cancer cells were seeded in 6 well plates at 1×10^5^ cells per well or 12 well plates at 5×10^4^ per well and allowed to adhere overnight and transfected with indicated concentrations (10μM, 20μM) of control (GE Dharmacon, CN-001000-01) or miR-29a (GE Dharmacon, C-300504-07) mimics, or 1μM siRNA using siCTRL (GE Dharmacon, D-001810-10-05), siTFEB (GE Dharmacon, L-009798-00-0005), and siATG9A (GE Dharmacon, L-014294-01-0005) using DharmaFECT®1 (GE Dharmacon, T-2001-01) as per the manufacturer's protocol. Total protein or RNA was isolated at 24 hours post-transfection for western blot or qPCR analysis respectively as described above.

### Migration and invasion measurements

1×10^4^ cells (Panc-1 or MIA PaCa-2) transfected with 20nM control or miR-29a mimics using DharmaFECT®1 were plated in triplicate in the upper chambers of 8μm transwells (Falcon, 353097) in 100μl serum-free media and 750μl 10% serum containing media in the lower chamber of 24-well plates and incubated at 37°C for 24 hours. For invasion assays, 80μl of 1:5 diluted matrigel (BD, 354234) was pre-coated in the upper chambers and allowed to solidify prior to plating cells. 24 hours post-seeding, membranes were washed twice with PBS, fixed with 4% paraformaldehyde, and stained with 0.1% crystal violet in 20% ethanol. Any cells remaining in the upper chamber were carefully removed, and cells migrated/invaded on to the lower membrane were imaged and counted. For each well, 5 random fields were counted, and average number of cells per field was presented.

### Measurements of cell viability, cytotoxicity, and caspase activity

5×10^3^ pancreatic cancer cells per well (Panc-1, MIA PaCa-2, or COLO 357) were plated in 96 well plates and grown at 37°C for 24 hours. Cells were then transfected with 20nM mimic control or miR-29a mimic using DharmaFECT®1 for 24 hours. Transfection media was then removed and replaced with complete media, and cells were allowed to recover for 24 hours and subsequently treated with varying concentrations of gemcitabine (0μM, 0.1μM, 1μM, 10μM, 100μM). Cell viability was measured at 72 hours post-gemcitabine treatment by adding 10ul Cell Counting Kit-8 (CCK8) reagent (Dojindo, CK04) and absorbance was measured at 450nm. For cell viability with Chloroquine (CQ) and BafilomycinA1 (BafA1) treatment, cells were treated with 25μM CQ (Sigma Aldrich, C6628) or 10μM BafA1 (Sigma Aldrich, B1793) in combination with 10μM gemcitabine for 48 hours, and viability was measured using CCK8 kit as described above. For cytotoxic effects and caspase activity, pancreatic cancer cells (Panc-1, MIA PaCa-2) were transfected with mimic control or miR-29a mimic as described above and treated with 10μM gemcitabine for 24-48 hours. For cytotoxic effects, lactate dehydrogenase release was determined using Promega CytoTox-ONE Homogeneous Membrane Integrity Assay (Promega, G7890) and fluorescence was measured at 560/590nm. Caspase activity was determined using Promega Apo-ONE Homogenous Caspase-3/7 Assay Kit (Promega, PRG7790) with fluorescence measured at 490/530nm.

### Soft agar assays

3×10^5^ pancreatic cancer cells per well (Panc-1 or MIA PaCa-2) were plated in 6 well plates and grown at 37°C for 24 hours. Cells were then transfected with 20nM mimic control or miR-29a mimic using DharmaFECT®. 1.5×10^3^ pancreatic cancer cells (Panc-1 or MIA PaCa-2) transfected with control or miR-29a mimics were plated per well in a 6 well plate containing 0.5% top agarose and 1% bottom agarose (BioRad, 162-0137). After 20 days, colonies were stained with crystal violet and were counted under low power bright field microscopy for positive colonies.

### Luciferase reporter assay

The 3′UTR containing predicted miR-29 binding sites, both wild type and mutant, for ATG9A and TFEB were cloned into pmirGLO Dual-Luciferase miRNA Target Expression Vector (Promega, #E1330) downstream of the firefly luciferase open reading frame. 5×10^3^ pancreatic cancer cells per well (Panc-1 or MIA PaCa-2) were plated in 96 well plates and grown at 37°C for 24 hours. Cells were then co-transfected for 24hrs with 10nM mimic control or miR-29a mimic and 100ng of pmirGLO Dual-Luciferase miRNA Target Expression Vector containing each respective 3′UTR binding site using DharmaFECT Duo Transfection Reagent (GE, T-2010-02). Cells were transfected for 24 hours, and luciferase levels were measured 24 hours post-transfection using Dual-Glo® Luciferase Assay System (Promega, #E2920). Firefly luciferase luminescence was normalized to renilla luciferase activity for each transfected well.

### Autophagy assays

For assessment of miR-29 effects on autophagy flux via immunoblotting, 1×10^5^ pancreatic cancer cells per well (Panc-1, MIA PaCa-2, or COLO 357) were plated in 12 well plates and grown at 37°C for 24 hours. Cells were then transfected with 10nM control or miR-29a mimics using DharmaFECT®1. 24 hours post-transfection, cells were treated with 25μM CQ in complete media for 3-6 hours. Subsequently, total proteins were harvested and subjected to western blot analysis as described above. Lentivirus encoding GFP-LC3B were generated using plasmid (GeneCopoepia, EX-T0824-Lv103) in HEK293 cells (ATCC, CRL-1573) via standard HEPES/Calcium Phosphate transfection. Stable Panc-1 GFP-LC3B cells were generated by transducing exponentially growing Panc-1 cells in T-75 flask. GFP positive cells were selected by flow cytometry and were expanded for one week prior to conducting experiments. For immunofluorescence imaging, cells were fixed with 4% PFA and permeabilized using 0.1% triton and blocked using 1% BSA. Primary LAMP-2 antibody (Santa Cruz, SC18822) was incubated overnight, followed by 1 hour secondary Alexa Fluor® 647 antibody (Abcam, ab150079) incubation and 10ug/mL Hoechst Nuclear Stain (Life Technologies, ab150083). Eight 0.5 micron Z-stack sections were captured using the Opera (Perkin Elmer) fluorescent microscope and final images were deconvolved and analyzed using Volocity imaging analysis software (Perkin Elmer). Quantifications for number of GFP-LC3B positive compartments and colocalization of GFP-LC3B and LAMP-2 were taken from 4 random fields with 8-10 cells per field.

### Statistics

ANOVA with Tukey's post-hoc test and 2-tailed Student's *t* tests were used to test for statistical significance. *P* < 0.05 was considered statistically significant.

## SUPPLEMENTARY FIGURES



## References

[1] Torre LA, Bray F, Siegel RL, Ferlay J, Lortet-Tieulent J, Jemal A (2015). Global cancer statistics, 2012. CA.

[2] Siegel RL, Miller KD, Jemal A (2016). Cancer statistics, 2016. CA.

[3] Rahib L, Smith BD, Aizenberg R, Rosenzweig AB, Fleshman JM, Matrisian LM (2014). Projecting cancer incidence and deaths to 2030: the unexpected burden of thyroid, liver, and pancreas cancers in the United States. Cancer Research.

[4] Von Hoff DD, Ervin T, Arena FP, Chiorean EG, Infante J, Moore M, Seay T, Tjulandin SA, Ma WW, Saleh MN, Harris M, Reni M, Dowden S (2013). Increased survival in pancreatic cancer with nab-paclitaxel plus gemcitabine. The New England Journal of Medicine.

[5] Conroy T, Desseigne F, Ychou M, Bouche O, Guimbaud R, Becouarn Y, Adenis A, Raoul JL, Gourgou-Bourgade S, de la Fouchardiere C, Bennouna J, Bachet JB, Khemissa-Akouz F (2011). FOLFIRINOX versus gemcitabine for metastatic pancreatic cancer. The New England Journal of Medicine.

[6] Ryan DP, Hong TS, Bardeesy N (2014). Pancreatic adenocarcinoma. The New England Journal of Medicine.

[7] He C, Klionsky DJ (2009). Regulation mechanisms and signaling pathways of autophagy. Annual review of genetics.

[8] Apel A, Herr I, Schwarz H, Rodemann HP, Mayer A (2008). Blocked autophagy sensitizes resistant carcinoma cells to radiation therapy. Cancer Research.

[9] Li J, Yang B, Zhou Q, Wu Y, Shang D, Guo Y, Song Z, Zheng Q, Xiong J (2013). Autophagy promotes hepatocellular carcinoma cell invasion through activation of epithelial-mesenchymal transition. Carcinogenesis.

[10] Fels DR, Ye J, Segan AT, Kridel SJ, Spiotto M, Olson M, Koong AC, Koumenis C (2008). Preferential cytotoxicity of bortezomib toward hypoxic tumor cells via overactivation of endoplasmic reticulum stress pathways. Cancer Research.

[11] Claerhout S, Verschooten L, Van Kelst S, De Vos R, Proby C, Agostinis P, Garmyn M (2010). Concomitant inhibition of AKT and autophagy is required for efficient cisplatin-induced apoptosis of metastatic skin carcinoma. International journal of cancer.

[12] Akalay I, Janji B, Hasmim M, Noman MZ, Andre F, De Cremoux P, Bertheau P, Badoual C, Vielh P, Larsen AK, Sabbah M, Tan TZ, Keira JH (2013). Epithelial-to-mesenchymal transition and autophagy induction in breast carcinoma promote escape from T-cell-mediated lysis. Cancer Research.

[13] Kanzawa T, Germano IM, Komata T, Ito H, Kondo Y, Kondo S (2004). Role of autophagy in temozolomide-induced cytotoxicity for malignant glioma cells. Cell Death Differ.

[14] Li M, Jiang X, Liu D, Na Y, Gao GF, Xi Z (2008). Autophagy protects LNCaP cells under androgen deprivation conditions. Autophagy.

[15] Yang A, Rajeshkumar NV, Wang X, Yabuuchi S, Alexander BM, Chu GC, Von Hoff DD, Maitra A, Kimmelman AC (2014). Autophagy is critical for pancreatic tumor growth and progression in tumors with p53 alterations. Cancer Discovery.

[16] Yang MC, Wang HC, Hou YC, Tung HL, Chiu TJ, Shan YS (2015). Blockade of autophagy reduces pancreatic cancer stem cell activity and potentiates the tumoricidal effect of gemcitabine. Molecular cancer.

[17] Yang S, Wang X, Contino G, Liesa M, Sahin E, Ying H, Bause A, Li Y, Stommel JM, Dell'antonio G, Mautner J, Tonon G, Haigis M (2011). Pancreatic cancers require autophagy for tumor growth. Genes & development.

[18] Hashimoto D, Blauer M, Hirota M, Ikonen NH, Sand J, Laukkarinen J (2014). Autophagy is needed for the growth of pancreatic adenocarcinoma and has a cytoprotective effect against anticancer drugs. Eur J Cancer.

[19] Donadelli M, Dando I, Zaniboni T, Costanzo C, Dalla Pozza E, Scupoli MT, Scarpa A, Zappavigna S, Marra M, Abbruzzese A, Bifulco M, Caraglia M, Palmieri M (2011). Gemcitabine/cannabinoid combination triggers autophagy in pancreatic Cancer Cells through a ROS-mediated mechanism. Cell Death Dis.

[20] Estes ML, Ewing-Wilson D, Chou SM, Mitsumoto H, Hanson M, Shirey E, Ratliff NB (1987). Chloroquine neuromyotoxicity. Clinical and pathologic perspective. The American journal of medicine.

[21] Ma X, Yan L, He L, He D, Lu H (2010). Ocular fundus manifestation of two patients following long-term chloroquine therapy: a case report. Diagnostic pathology.

[22] Nogueira HM, Gama RD (2009). Images in clinical medicine. Bull's-eye maculopathy. The New England Journal of Medicine.

[23] Veinot JP, Mai KT, Zarychanski R (1998). Chloroquine related cardiac toxicity. The Journal of rheumatology.

[24] Tonnesmann E, Kandolf R, Lewalter T (2013). Chloroquine cardiomyopathy - a review of the literature. Immunopharmacology and immunotoxicology.

[25] Janssen HL, Reesink HW, Lawitz EJ, Zeuzem S, Rodriguez-Torres M, Patel K, van der Meer AJ, Patick AK, Chen A, Zhou Y, Persson R, King BD, Kauppinen S (2013). Treatment of HCV infection by targeting microRNA. The New England Journal of Medicine.

[26] Chau BN, Xin C, Hartner J, Ren S, Castano AP, Linn G, Li J, Tran PT, Kaimal V, Huang X, Chang AN, Li S, Kalra A (2012). MicroRNA-21 promotes fibrosis of the kidney by silencing metabolic pathways. Science translational medicine.

[27] Kasinski AL, Kelnar K, Stahlhut C, Orellana E, Zhao J, Shimer E, Dysart S, Chen X, Bader AG, Slack FJ (2015). A combinatorial microRNA therapeutics approach to suppressing non-small cell lung cancer. Oncogene.

[28] Xue W, Dahlman JE, Tammela T, Khan OF, Sood S, Dave A, Cai W, Chirino LM, Yang GR, Bronson R, Crowley DG, Sahay G, Schroeder A (2014). Small RNA combination therapy for lung cancer. Proceedings of the National Academy of Sciences of the United States of America.

[29] Krzeszinski JY, Wei W, Huynh H, Jin Z, Wang X, Chang TC, Xie XJ, He L, Mangala LS, Lopez-Berestein G, Sood AK, Mendell JT, Wan Y (2014). miR-34a blocks osteoporosis and bone metastasis by inhibiting osteoclastogenesis and Tgif2. Nature.

[30] Daige CL, Wiggins JF, Priddy L, Nelligan-Davis T, Zhao J, Brown D (2014). Systemic delivery of a miR34a mimic as a potential therapeutic for liver cancer. Molecular Cancer Therapeutics.

[31] Pramanik D, Campbell NR, Karikari C, Chivukula R, Kent OA, Mendell JT, Maitra A (2011). Restitution of tumor suppressor microRNAs using a systemic nanovector inhibits pancreatic cancer growth in mice. Molecular Cancer Therapeutics.

[32] Lee RC, Feinbaum RL, Ambros V (1993). The C. elegans heterochronic gene lin-4 encodes small RNAs with antisense complementarity to lin-14. Cell.

[33] Wightman B, Ha I, Ruvkun G (1993). Posttranscriptional regulation of the heterochronic gene lin-14 by lin-4 mediates temporal pattern formation in C. elegans. Cell.

[34] Bader AG, Brown D, Winkler M (2010). The promise of microRNA replacement therapy. Cancer Research.

[35] Kota J, Chivukula RR, O'Donnell KA, Wentzel EA, Montgomery CL, Hwang HW, Chang TC, Vivekanandan P, Torbenson M, Clark KR, Mendell JR, Mendell JT (2009). Therapeutic microRNA delivery suppresses tumorigenesis in a murine liver cancer model. Cell.

[36] Hsu SH, Wang B, Kota J, Yu J, Costinean S, Kutay H, Yu L, Bai S, La Perle K, Chivukula RR, Mao H, Wei M, Clark KR (2012). Essential metabolic, anti-inflammatory, and anti-tumorigenic functions of miR-122 in liver. The Journal of Clinical Investigation.

[37] Zou Y, Li J, Chen Z, Li X, Zheng S, Yi D, Zhong A, Chen J (2015). miR-29c suppresses pancreatic cancer liver metastasis in an orthotopic implantation model in nude mice and affects survival in pancreatic cancer patients. Carcinogenesis.

[38] Trehoux S, Lahdaoui F, Delpu Y, Renaud F, Leteurtre E, Torrisani J, Jonckheere N, Van Seuningen I (2015). Micro-RNAs miR-29a and miR-330-5p function as tumor suppressors by targeting the MUC1 mucin in pancreatic Cancer Cells. Biochimica et biophysica acta.

[39] Kwon JJ, Nabinger SC, Vega Z, Sahu SS, Alluri RK, Abdul-Sater Z, Yu Z, Gore J, Nalepa G, Saxena R, Korc M, Kota J (2015). Pathophysiological role of microRNA-29 in pancreatic cancer stroma. Scientific Reports.

[40] Long J, Zhang Y, Yu X, Yang J, LeBrun DG, Chen C, Yao Q, Li M (2011). Overcoming drug resistance in pancreatic cancer. Expert opinion on therapeutic targets.

[41] Olive KP, Jacobetz MA, Davidson CJ, Gopinathan A, McIntyre D, Honess D, Madhu B, Goldgraben MA, Caldwell ME, Allard D, Frese KK, Denicola G, Feig C (2009). Inhibition of Hedgehog signaling enhances delivery of chemotherapy in a mouse model of pancreatic cancer. Science.

[42] Pan X, Arumugam T, Yamamoto T, Levin PA, Ramachandran V, Ji B, Lopez-Berestein G, Vivas-Mejia PE, Sood AK, McConkey DJ, Logsdon CD (2008). Nuclear factor-kappaB p65/relA silencing induces apoptosis and increases gemcitabine effectiveness in a subset of pancreatic Cancer Cells. Clinical Cancer Research.

[43] Arnold NB, Arkus N, Gunn J, Korc M (2007). The histone deacetylase inhibitor suberoylanilide hydroxamic acid induces growth inhibition and enhances gemcitabine-induced cell death in pancreatic cancer. Clinical Cancer Research.

[44] Korzeniewski C, Callewaert DM (1983). An enzyme-release assay for natural cytotoxicity. Journal of immunological methods.

[45] Decker T, Lohmann-Matthes ML (1988). A quick and simple method for the quantitation of lactate dehydrogenase release in measurements of cellular cytotoxicity and tumor necrosis factor (TNF) activity. Journal of immunological methods.

[46] Mann SS, Hammarback JA (1994). Molecular characterization of light chain 3. A microtubule binding subunit of MAP1A and MAP1B. The Journal of Biological Chemistry.

[47] Ichimura Y, Kirisako T, Takao T, Satomi Y, Shimonishi Y, Ishihara N, Mizushima N, Tanida I, Kominami E, Ohsumi M, Noda T, Ohsumi Y (2000). A ubiquitin-like system mediates protein lipidation. Nature.

[48] Kabeya Y, Mizushima N, Ueno T, Yamamoto A, Kirisako T, Noda T, Kominami E, Ohsumi Y, Yoshimori T (2000). LC3, a mammalian homologue of yeast Apg8p, is localized in autophagosome membranes after processing. The EMBO journal.

[49] He H, Dang Y, Dai F, Guo Z, Wu J, She X, Pei Y, Chen Y, Ling W, Wu C, Zhao S, Liu JO, Yu L (2003). Post-translational modifications of three members of the human MAP1LC3 family and detection of a novel type of modification for MAP1LC3B. The Journal of Biological Chemistry.

[50] Tanida I, Ueno T, Kominami E (2004). Human light chain 3/MAP1LC3B is cleaved at its carboxyl-terminal Met121 to expose Gly120 for lipidation and targeting to autophagosomal membranes. The Journal of Biological Chemistry.

[51] Wu J, Dang Y, Su W, Liu C, Ma H, Shan Y, Pei Y, Wan B, Guo J, Yu L (2006). Molecular cloning and characterization of rat LC3A and LC3B--two novel markers of autophagosome. Biochemical and biophysical research communications.

[52] Zhang XJ, Chen S, Huang KX, Le WD (2013). Why should autophagic flux be assessed?. Acta pharmacologica Sinica.

[53] Mizushima N, Yoshimori T (2007). How to interpret LC3 immunoblotting. Autophagy.

[54] Poole B, Ohkuma S (1981). Effect of weak bases on the intralysosomal pH in mouse peritoneal macrophages. The Journal of Cell Biology.

[55] Shintani T, Klionsky DJ (2004). Autophagy in health and disease: a double-edged sword. Science.

[56] van Schalkwyk DA, Chan XW, Misiano P, Gagliardi S, Farina C, Saliba KJ (2010). Inhibition of Plasmodium falciparum pH regulation by small molecule indole derivatives results in rapid parasite death. Biochemical pharmacology.

[57] Mauvezin C, Nagy P, Juhasz G, Neufeld TP (2015). Autophagosome-lysosome fusion is independent of V-ATPase-mediated acidification. Nature communications.

[58] Mukubou H, Tsujimura T, Sasaki R, Ku Y (2010). The role of autophagy in the treatment of pancreatic cancer with gemcitabine and ionizing radiation. International journal of oncology.

[59] Donohue E, Thomas A, Maurer N, Manisali I, Zeisser-Labouebe M, Zisman N, Anderson HJ, Ng SS, Webb M, Bally M, Roberge M (2013). The autophagy inhibitor verteporfin moderately enhances the antitumor activity of gemcitabine in a pancreatic ductal adenocarcinoma model. Journal of Cancer.

[60] Settembre C, Di Malta C, Polito VA, Garcia Arencibia M, Vetrini F, Erdin S, Erdin SU, Huynh T, Medina D, Colella P, Sardiello M, Rubinsztein DC, Ballabio A (2011). TFEB links autophagy to lysosomal biogenesis. Science.

[61] Argani P (2015). MiT family translocation renal cell carcinoma. Seminars in diagnostic pathology.

[62] Giatromanolaki A, Sivridis E, Mitrakas A, Kalamida D, Zois CE, Haider S, Piperidou C, Pappa A, Gatter KC, Harris AL, Koukourakis MI (2014). Autophagy and lysosomal related protein expression patterns in human glioblastoma. Cancer Biol Ther.

[63] Perera RM, Stoykova S, Nicolay BN, Ross KN, Fitamant J, Boukhali M, Lengrand J, Deshpande V, Selig MK, Ferrone CR, Settleman J, Stephanopoulos G, Dyson NJ (2015). Transcriptional control of autophagy-lysosome function drives pancreatic cancer metabolism. Nature.

[64] Klionsky DJ, Cregg JM, Dunn WA, Emr SD, Sakai Y, Sandoval IV, Sibirny A, Subramani S, Thumm M, Veenhuis M, Ohsumi Y (2003). A unified nomenclature for yeast autophagy-related genes. Developmental Cell.

[65] Yen WL, Klionsky DJ (2007). Atg27 is a second transmembrane cycling protein. Autophagy.

[66] Young AR, Chan EY, Hu XW, Kochl R, Crawshaw SG, High S, Hailey DW, Lippincott-Schwartz J, Tooze SA (2006). Starvation and ULK1-dependent cycling of mammalian Atg9 between the TGN and endosomes. Journal of cell science.

[67] Orsi A, Razi M, Dooley HC, Robinson D, Weston AE, Collinson LM, Tooze SA (2012). Dynamic and transient interactions of Atg9 with autophagosomes, but not membrane integration, are required for autophagy. Molecular Biology of the Cell.

[68] Tang JY, Hsi E, Huang YC, Hsu NC, Chen YK, Chu PY, Chai CY (2013). ATG9A overexpression is associated with disease recurrence and poor survival in patients with oral squamous cell carcinoma. Virchows Archiv : an international journal of pathology.

[69] Dai F, Zhang Y, Chen Y (2014). Involvement of miR-29b signaling in the sensitivity to chemotherapy in patients with ovarian carcinoma. Human pathology.

[70] Ru P, Steele R, Newhall P, Phillips NJ, Toth K, Ray RB (2012). miRNA-29b suppresses prostate cancer metastasis by regulating epithelial-mesenchymal transition signaling. Molecular Cancer Therapeutics.

[71] Wang B, Li W, Liu H, Yang L, Liao Q, Cui S, Wang H, Zhao L (2014). miR-29b suppresses tumor growth and metastasis in colorectal cancer via downregulating Tiam1 expression and inhibiting epithelial-mesenchymal transition. Cell death & disease.

[72] Chou J, Lin JH, Brenot A, Kim JW, Provot S, Werb Z (2013). GATA3 suppresses metastasis and modulates the tumour microenvironment by regulating microRNA-29b expression. Nature Cell Biology.

[73] Yang AD, Camp ER, Fan F, Shen L, Gray MJ, Liu W, Somcio R, Bauer TW, Wu Y, Hicklin DJ, Ellis LM (2006). Vascular endothelial growth factor receptor-1 activation mediates epithelial to mesenchymal transition in human pancreatic carcinoma cells. Cancer Research.

[74] Rasheed ZA, Yang J, Wang Q, Kowalski J, Freed I, Murter C, Hong SM, Koorstra JB, Rajeshkumar NV, He X, Goggins M, Iacobuzio-Donahue C, Berman DM (2010). Prognostic significance of tumorigenic cells with mesenchymal features in pancreatic adenocarcinoma. Journal of the National Cancer Institute.

[75] Hotz B, Arndt M, Dullat S, Bhargava S, Buhr HJ, Hotz HG (2007). Epithelial to mesenchymal transition: expression of the regulators snail, slug, and twist in pancreatic cancer. Clinical Cancer Research.

[76] Reggiori F, Tooze SA (2012). Autophagy regulation through Atg9 traffic. The Journal of Cell Biology.

[77] Webber JL, Young AR, Tooze SA (2007). Atg9 trafficking in Mammalian cells. Autophagy.

[78] Mott JL, Kobayashi S, Bronk SF, Gores GJ (2007). mir-29 regulates Mcl-1 protein expression and apoptosis. Oncogene.

[79] Lamouille S, Xu J, Derynck R (2014). Molecular mechanisms of epithelial-mesenchymal transition. Nature reviews Molecular cell biology.

[80] Taddei ML, Giannoni E, Fiaschi T, Chiarugi P (2012). Anoikis: an emerging hallmark in health and diseases. The Journal of pathology.

[81] Frisch SM, Francis H (1994). Disruption of epithelial cell-matrix interactions induces apoptosis. The Journal of Cell Biology.

[82] Horbinski C, Mojesky C, Kyprianou N (2010). Live free or die: tales of homeless (cells) in cancer. Am J Pathol.

[83] Sun C, Yamato T, Furukawa T, Ohnishi Y, Kijima H, Horii A (2001). Characterization of the mutations of the K-ras, p53, p16, and SMAD4 genes in 15 human pancreatic Cancer Cell lines. Oncology reports.

[84] Massague J (2012). TGFbeta signalling in context. Nature reviews Molecular cell biology.

[85] Noetel A, Kwiecinski M, Elfimova N, Huang J, Odenthal M (2012). microRNA are Central Players in Anti- and Profibrotic Gene Regulation during Liver Fibrosis. Frontiers in physiology.

[86] Mott JL, Kurita S, Cazanave SC, Bronk SF, Werneburg NW, Fernandez-Zapico ME (2010). Transcriptional suppression of mir-29b-1/mir-29a promoter by c-Myc, hedgehog, and NF-kappaB. Journal of Cellular Biochemistry.

[87] Schild C, Wirth M, Reichert M, Schmid RM, Saur D, Schneider G (2009). PI3K signaling maintains c-myc expression to regulate transcription of E2F1 in pancreatic Cancer Cells. Molecular carcinogenesis.

[88] Rajurkar M, De Jesus-Monge WE, Driscoll DR, Appleman VA, Huang H, Cotton JL, Klimstra DS, Zhu LJ, Simin K, Xu L, McMahon AP, Lewis BC, Mao J (2012). The activity of Gli transcription factors is essential for Kras-induced pancreatic tumorigenesis. Proceedings of the National Academy of Sciences of the United States of America.

[89] Ding ZB, Hui B, Shi YH, Zhou J, Peng YF, Gu CY, Yang H, Shi GM, Ke AW, Wang XY, Song K, Dai Z, Shen YH (2011). Autophagy activation in hepatocellular carcinoma contributes to the tolerance of oxaliplatin via reactive oxygen species modulation. Clinical Cancer Research.

[90] Yoon JH, Ahn SG, Lee BH, Jung SH, Oh SH (2012). Role of autophagy in chemoresistance: regulation of the ATM-mediated DNA-damage signaling pathway through activation of DNA-PKcs and PARP-1. Biochemical pharmacology.

[91] Sui X, Chen R, Wang Z, Huang Z, Kong N, Zhang M, Han W, Lou F, Yang J, Zhang Q, Wang X, He C, Pan H (2013). Autophagy and chemotherapy resistance: a promising therapeutic target for cancer treatment. Cell death & disease.

[92] Morgan RT, Woods LK, Moore GE, Quinn LA, McGavran L, Gordon SG (1980). Human cell line (COLO 357) of metastatic pancreatic adenocarcinoma. International journal of cancer.

