# Supplementary material for: Novel role of miR-29a in pancreatic cancer autophagy and its therapeutic potential
Source: Oncotarget. 2016 Sep 10;7(44):71635–50. doi: 10.18632/oncotarget.11928 (PMC5342107; doi:10.18632/oncotarget.11928)
Supplement: Supplementary file 1 [file oncotarget-07-71635-s001.pdf]

Novel role of miR-29a in pancreatic cancer autophagy and its therapeutic potential

SUPPLEMENTARY FIGURES

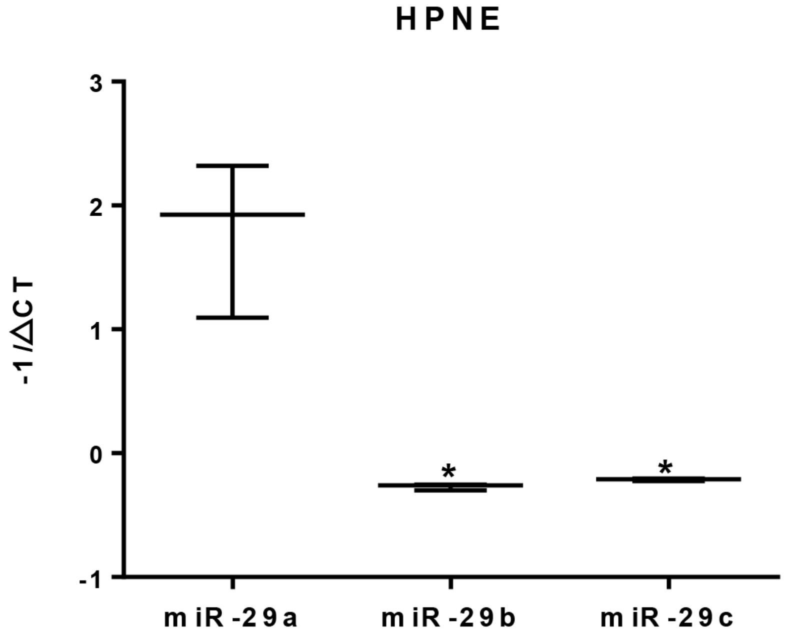

Supplementary Figure S1: miR-29a is the most abundantly expressed miR-29 family member in human pancreatic normal epithelial cell line. RNA was isolated from human pancreatic normal epithelial cells (HPNE) (n=3) and miR-29a, -29b, and -29c expression levels were determined by qPCR analysis using U6 snRNA as an internal control. Delta CT (ΔCT) was calculated for each miR-29 family member to measure relative expression levels. Boxplots represent -1/ΔCT of miR-29 family expression levels. *$p<0.05$.

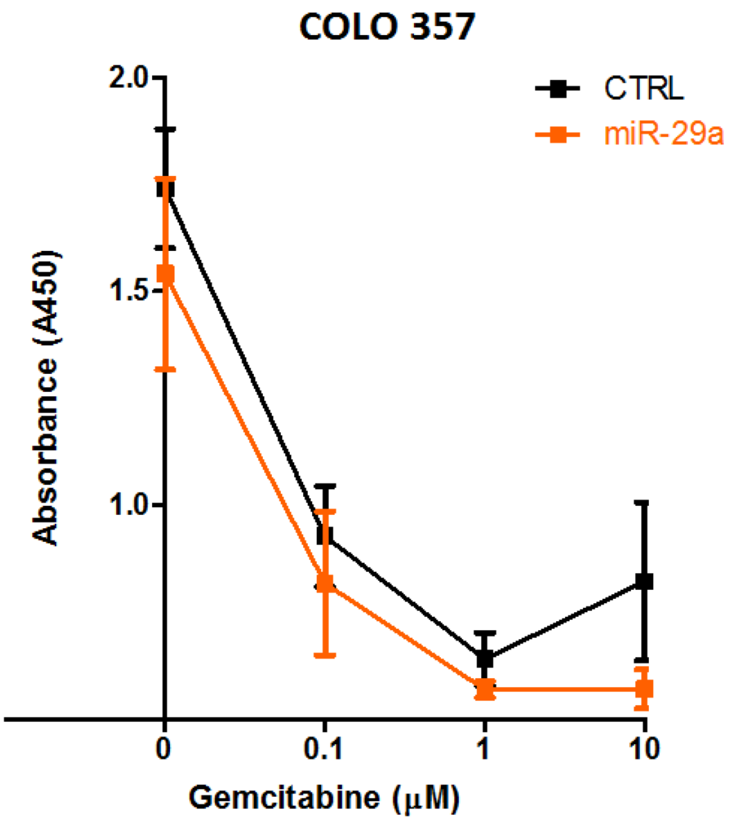

Supplementary Figure S2: Effect of miR-29a overexpression and gemcitabine treatment on COLO 357 cell viability. COLO 357 were seeded into a 96-well plate, transfected for 24hrs with CTRL or miR-29a mimics, and treated with various concentrations of gemcitabine, and cell viability was measured at 72 hours post-treatment using the Cell Counting Kit-8 (CCK-8) assay according to manufacture's protocol. Average relative absorbance (A450) is presented (n=6) ± Standard Error of the Mean (S.E.M.).

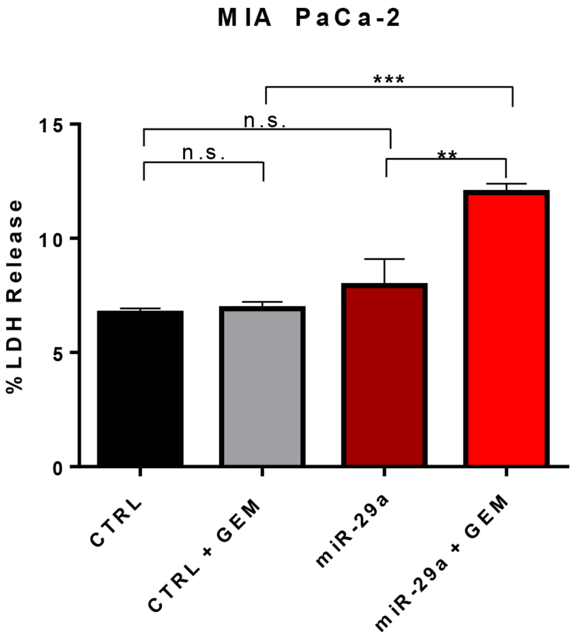

Supplementary Figure S3: LDH release in MIA PaCa-2 cells treated with gemcitabine in combination with miR-29a. MIA PaCa-2 cells were transfected with CTRL or miR-29a mimics, treated with gemcitabine (GEM) for 48 hours and lactate dehydrogenase (LDH) release was determined by substrate based activity assay (fluorescence 560/590nm). Average relative percent cytotoxicity are represented (n=4) ± S.E.M.

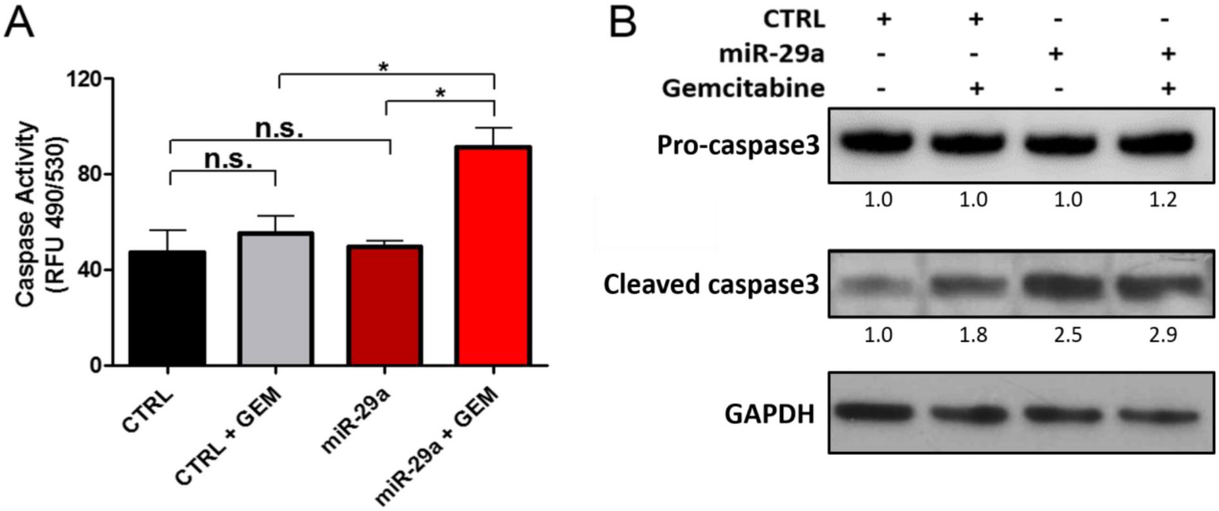

Supplementary Figure S4: Caspase3/7 activity and activated caspase 3 levels in MIA PaCa-2 cells treated with gemcitabine in combination with miR-29a. MIA PaCa-2 cells were transfected with CTRL or miR-29a mimics. **A.** Transfected cells were treated with 10µM gemcitabine (GEM) for 24 hours, lysed, and caspase activity was determined using Apo-ONE Homogeneous Caspase-3/7 Assay. Average fluorescence (490/530nm) are represented (n=4) ± S.E.M. **B.** Transfected cells were treated with 10µM GEM for 12 hours. Post GEM treatment, 15ug of total protein lysate was subjected to western blot analysis for pro-caspase 3 and cleaved caspase 3 quantification. GAPDH was used as loading control. Relative quantification of band intensities normalized to GAPDH are shown below respective blots.

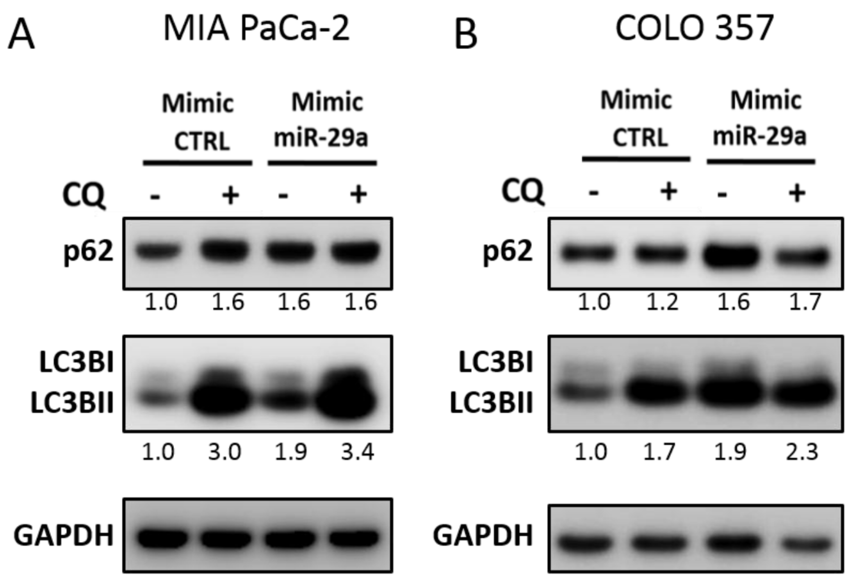

Supplementary Figure S5: miR-29a overexpression inhibits autophagic flux in MIA PaCa-2 and COLO 357 cells. **A.** MIA PaCa-2 or **B.** COLO 357 pancreatic cancer cells were transfected with CTRL or miR-29a mimics. 24hrs post-transfection, cells were treated with 25µM Chloroquine (CQ) for 3 hours and total protein was harvested. 5ug of total protein lysate was subjected to western blot analysis for p62 and LC3B quantification. GAPDH was used as loading control. Relative quantification of band intensities normalized to GAPDH are shown below respective blots.

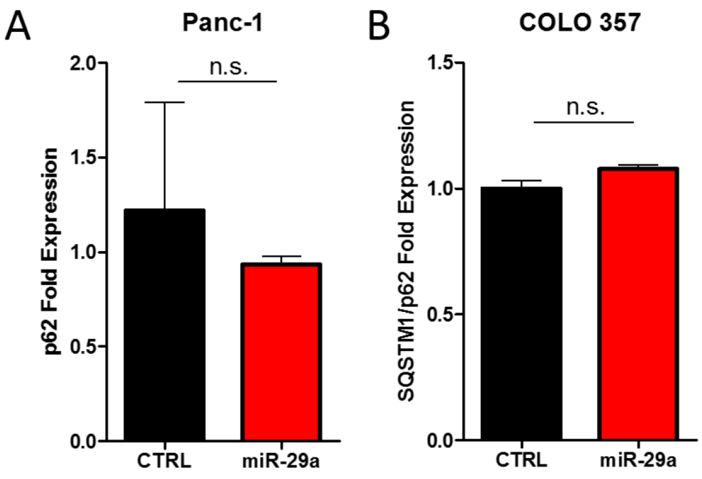

Supplementary Figure S6: p62 transcriptional expression is unaltered by miR-29a overexpression in Panc-1 and COLO 357 cells. **A.** Panc-1 or **B.** COLO 357 pancreatic cancer cells were transfected with CTRL or miR-29a mimics (n=3). 24hrs post-transfection, total RNA was harvested and subjected to qPCR analysis of p62. ACTB was used as an endogenous control. Data represented as average fold change ($^{\Delta\Delta}$CT) ± S.E.M.

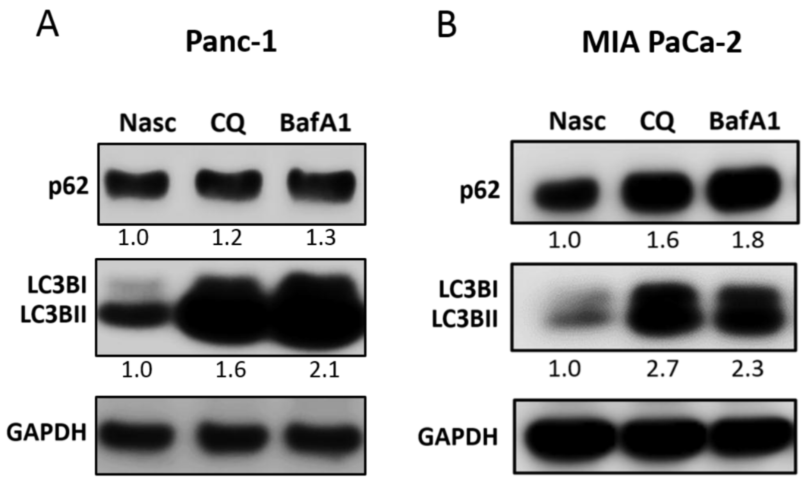

Supplementary Figure S7: Effect of CQ and BafA1 on Panc-1 and MIA PaCa-2 autophagy. A. Panc-1 or **B.** MIA PaCa-2 cells were treated with either 25µM Chloroquine (CQ) or 10µM BafilomycinA1 (BafA1) for 6hrs and lysed. 5ug of total protein lysate was subjected to western blot analysis for p62 and LC3B quantification. GAPDH was used as loading control. Relative quantification of band intensities normalized to GAPDH are shown below respective blots.

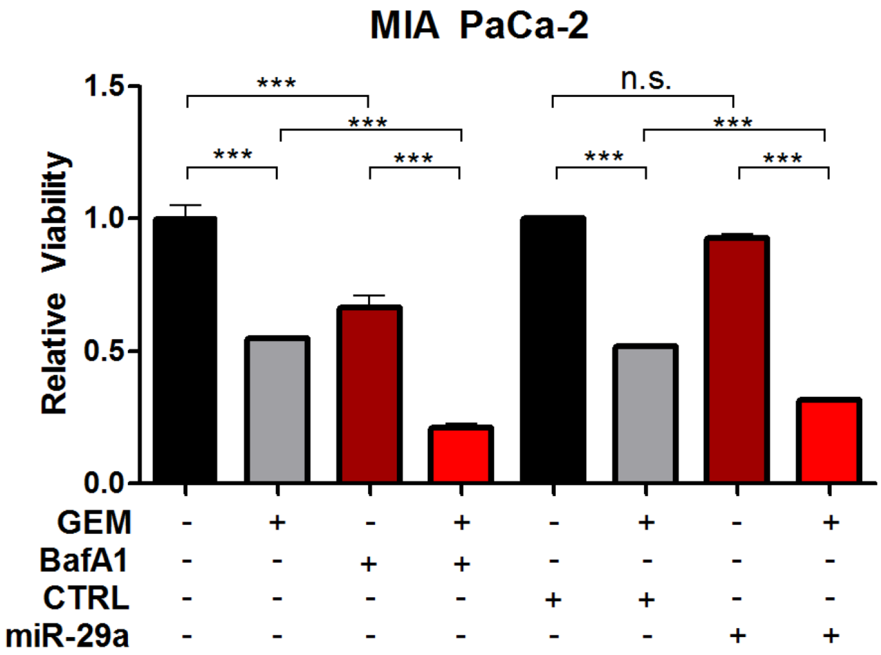

Supplementary Figure S8: Effect of miR-29a, CQ, and BafA1 on sensitization of MIA PaCa-2 cells to gemcitabine treatment. MIA PaCa-2 cells were transfected with CTRL or miR-29a mimics and treated with 10µM gemcitabine (GEM). In parallel, MIA PaCa-2 cells were treated with 10µM GEM alone or in combination with 25µM CQ or 10µM BafA1. 48 hours post GEM treatment, viability was determined using CCK-8 assay kit. Average relative absorbance (A450) normalized to respective controls is presented (N=6) ± S.E.M. ***p<0.001.

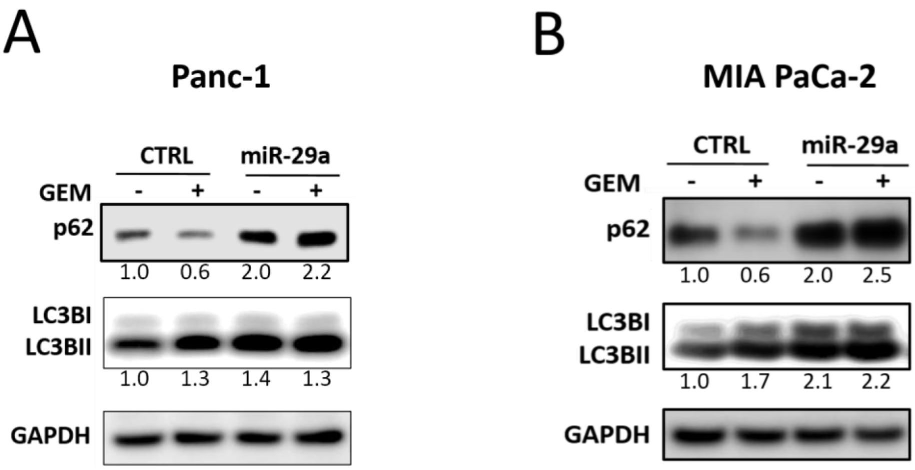

Supplementary Figure S9: miR-29a blocks gemcitabine induced autophagy in Panc-1 and MIA PaCa-2. A. Panc-1 or **B.** MIA PaCa-2 cells transfected with CTRL or miR-29a mimics, were treated with or without 10µM GEM and 5ug of total cell lysates were subjected to western blot analysis for p62 and LC3B quantification. GAPDH was used as loading control. Relative quantification of band intensities normalized to GAPDH are shown below respective blots.

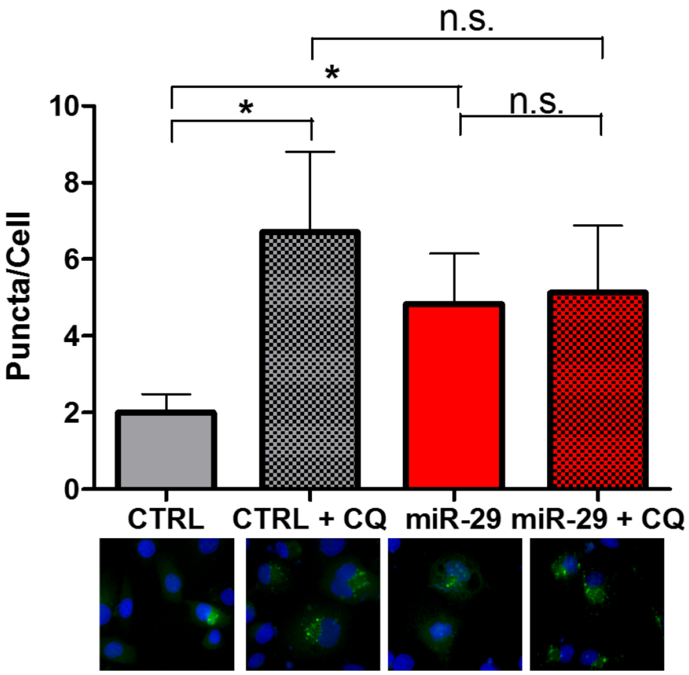

Supplementary Figure S10: LC3B puncta quantification in miR-29a overexpressing Panc-1 cells. GFP-LC3B stable Panc-1 cells were transfected with CTRL or miR-29a mimics. Following transfection, cells were treated with 25µM CQ for 3 hours, fixed, and imaged. Number of GFP-LC3B positive puncta was counted and average number of GFP-LC3B positive puncta per cell (N>25 cells/group) is presented ± S.E.M (n=3). Representative images are shown below each graph. *p<0.05, non-significant (n.s.).

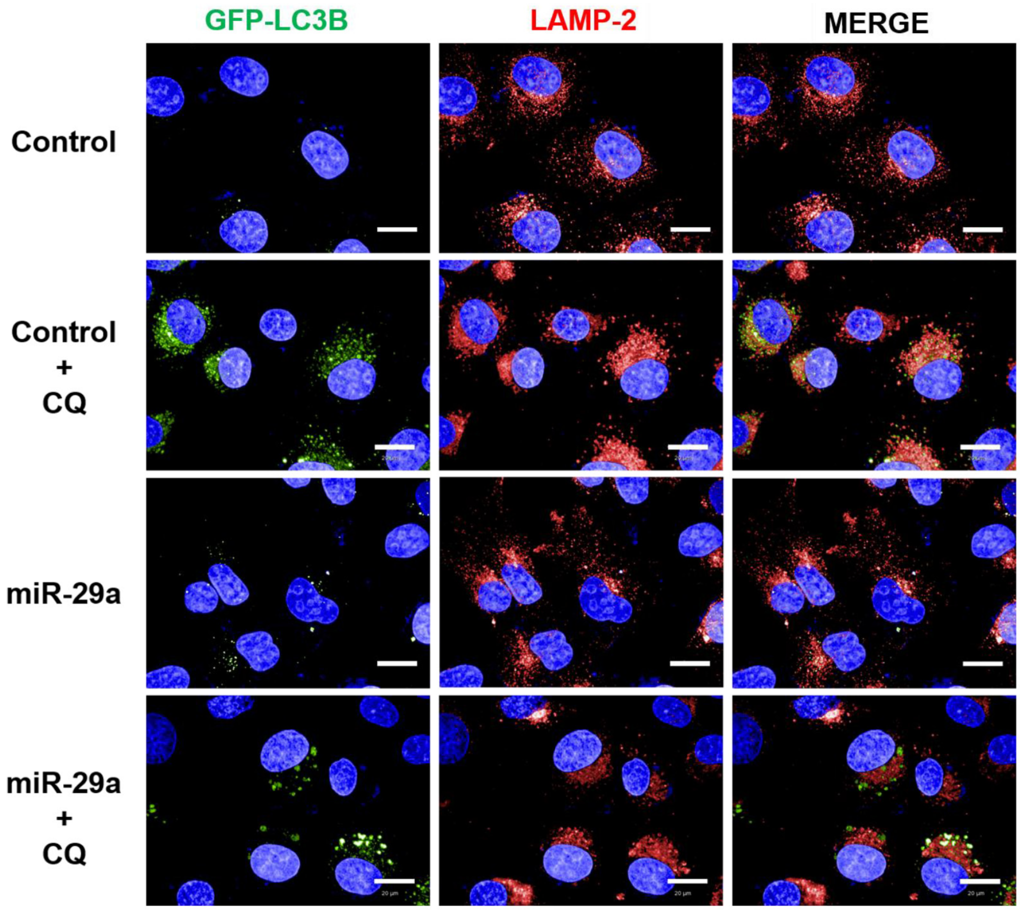

Supplementary Figure S11: 2D representative images of LC3B and LAMP-2 colocalization in miR-29a overexpressing Panc-1 cells. GFP-LC3B stable Panc-1 cells were transfected with CTRL or miR-29a mimics. Following transfection, cells were treated with 25µM CQ for 3 hours, fixed, and stained for LAMP-2, lysosomal marker (60x magnification). Scale bar indicates 20µm.

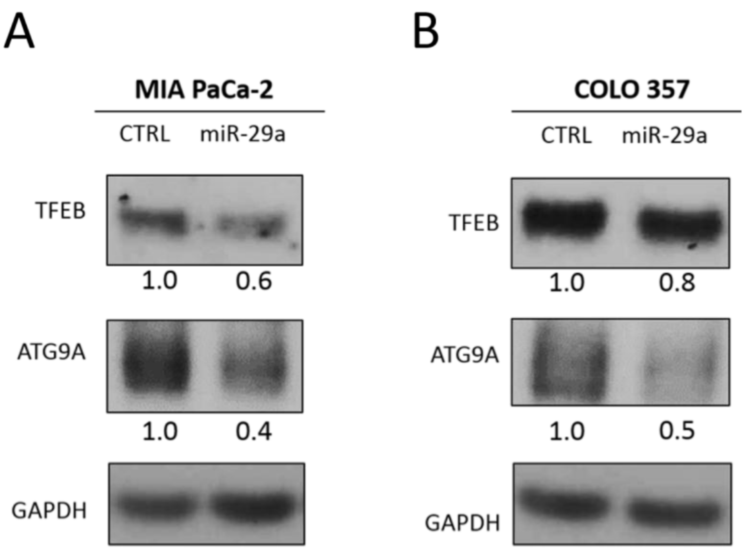

Supplementary Figure S12: Effect of miR-29a overexpression on TFEB and ATG9A expression in MIA PaCa-2 and COLO 357 cells. A. MIA PaCa-2 and **B.** COLO 357 cells transfected with CTRL or miR-29a mimics. 10ug total protein lysate was subjected to western blot analysis for ATG9A and TFEB quantification. GAPDH was used as a loading control. Relative quantification of band intensities normalized to GAPDH are shown below respective blots.

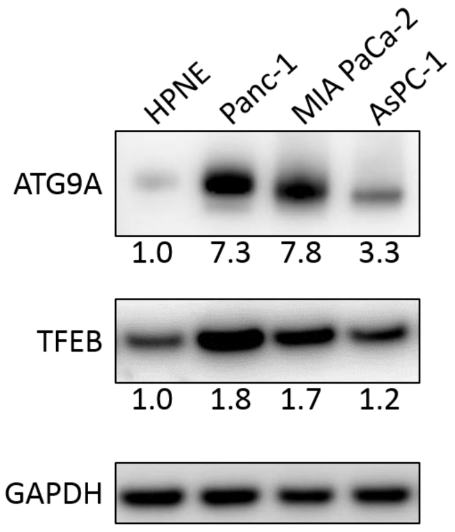

Supplementary Figure S13: ATG9A and TFEB expression in HPNE, Panc-1, MIA PaCa-2, and AsPC-1. Total protein lysates (10ug) harvested from normal epithelial cells, HPNE, and cancer cell lines, Panc-1, MIA PaCa-2, and AsPC-1, were subjected to western blot analysis for ATG9A and TFEB quantification. GAPDH was used as loading control. Relative quantification of band intensities normalized to GAPDH are shown below respective blots.

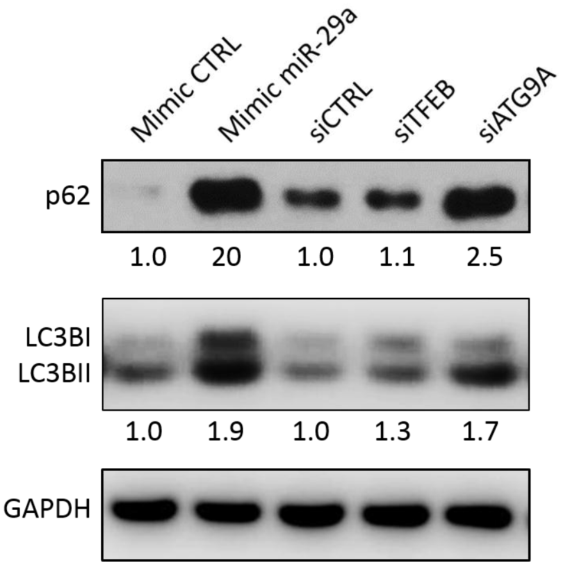

Supplementary Figure S14: Effect of TFEB and ATG9A knockdown on autophagy of MIA PaCa-2 cells. MIA PaCa-2 cells were transfected with CTRL miR-29a mimics, or with siCTRL, siTFEB, siATG9A. 24 hours post-transfection, total protein was harvested and 5ug of total protein lysate was subjected to western blot analysis for p62 and LC3B quantification. GAPDH was used as loading control. Relative quantification of band intensities normalized to respective controls are shown below respective blots.

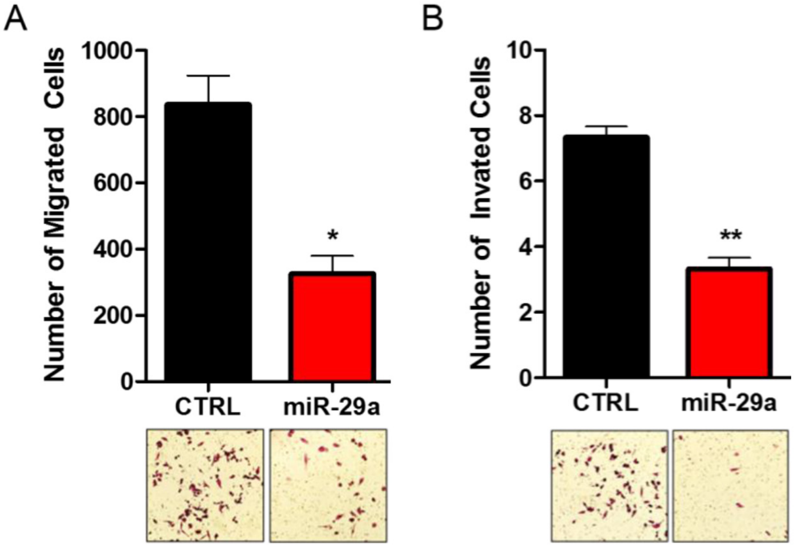

Supplementary Figure S15: Effect of miR-29a overexpression on migration and invasion of MIA PaCa-2 cells. MIA PaCa-2 cells were transfected with control (CTRL) or miR-29a mimics and plated into **A.** migration and **B.** invasion assays. Migration and invasion data presented as average number of cells per 5 fields (n=3) ± S.E.M. with representative images below each graph. *p<0.05, **p<0.01.

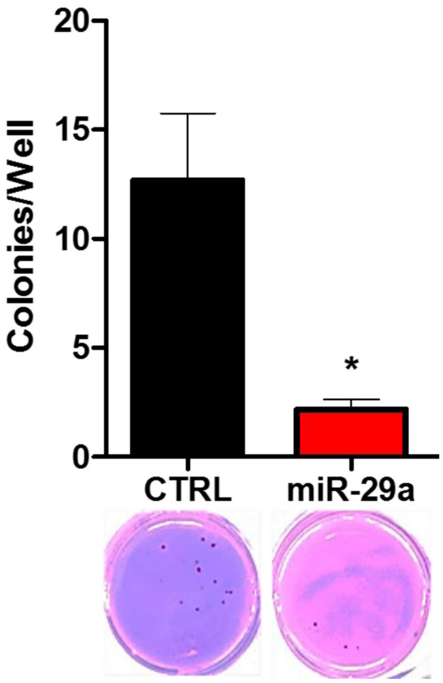

Supplementary Figure S16: Effect of miR-29a overexpression on anchorage independent growth of MIA PaCa-2 cells. MIA PaCa-2 cells were transfected with control (CTRL) or miR-29a mimics and plated into soft agar assays. Representative data is presented as average ± S.E.M. (n=3). Representative images are below each graph. *$p<0.05$.
